# The emergence of carbapenem resistant *Klebsiella pneumoniae* in Malaysia: correlation between microbiological trends with host characteristics and clinical factors

**DOI:** 10.1186/s13756-016-0164-x

**Published:** 2017-01-07

**Authors:** Ye-Mun Low, Polly Soo-Xi Yap, Kartini Abdul Jabar, Sasheela Ponnampalavanar, Rina Karunakaran, Rukumani Velayuthan, Chun-Wie Chong, Sazaly Abu Bakar, Mohd Yasim Md Yusof, Cindy Shuan-Ju Teh

**Affiliations:** 1 Department of Medical Microbiology, University of Malaya, 50603 Kuala Lumpur, Malaysia; 2 Department of Medicine, University of Malaya, 50603 Kuala Lumpur, Malaysia; 3 Department of Life Sciences, International Medical University, Bukit Jalil, 57000 Kuala Lumpur, Malaysia; 4 The Tropical Infectious Diseases Research & Education Centre (TIDREC), University of Malaya, 50603 Kuala Lumpur, Malaysia

**Keywords:** Carbapenem resistant *K. pneumoniae*, OXA-48, KPC-2, ST101, Enterobacteriaceae

## Abstract

**Background:**

Carbapenem resistant Enterobacteriaceae is a growing concern worldwide including Malaysia. The emergence of this pathogen is worrying because carbapenem is one of the 'last-line' antibiotics. The main objective of this study was to determine the prevalence of genetic mechanisms and clinical risk factors of carbapenem resistant *Klebsiella pneumoniae (K. pneumoniae)* in Malaysia.

**Methods:**

In this study, seventeen carbapenem resistant *K. pneumoniae* strains isolated from a tertiary teaching hospital in 2013 were studied. Minimal inhibitory concentration (MIC) of the bacterial strains was determined and genes associated with carbapenemases and extended-spectrum-beta-lactamases (ESBLs) were sequenced and compared with the closest representatives published in public domains. All strains were also sub-typed using pulsed-field gel electrophoresis (PFGE) and multilocus sequence typing (MLST). Statistical analyses were performed to determine the correlation between risk factors for acquiring carbapenem resistant *K. pneumoniae* and in-hospital mortality.

**Results:**

The predominant carbapenemase was *bla*_OXA-48_, detected in 12 strains (70.59%). Other carbapenemases detected in this study were *bla*_KPC-2_, *bla*_IMP-8_, *bla*_NMC-A_ and *bla*_NDM-1_. Nine different pulsotypes were identified and nine strains which were affiliated with ST101, the predominant sequence type had similar PFGE patterns (similarity index of 85%). Based on univariate statistical analysis, resistance to imipenem and usage of mechanical ventilation showed a statistically significant effect separately to in-hospital mortality.

**Conclusion:**

The diverse genetic mechanisms harbored by these carbapenem resistant *K. pneumoniae* facilitates its spread and complicates its detection. Thus, correlation between microbiological trends with host characteristics and clinical factors will provide a better insight of rational treatment strategies and pathogen control.

## Background

*Klebsiella pneumoniae (K. pneumoniae)* is an important pathogen responsible for many healthcare associated infections. This Gram-negative bacterium affiliated with the Enterobacteriaceae family is the fourth and fifth most common cause of pneumonia and bacteremia respectively, among intensive care units (ICU), newborn units and in immunocompromised patients [1, 2]. In hospital environments, *Klebsiella* species survive and multiply in wet environmental sites and colonize the human bowel, bladder, upper respiratory tract and skin [3].

During the 1990s, extended-spectrum beta-lactamases (ESBLs) producing *Klebsiella* species that are able to hydrolyze broad and extended-spectrum cephalosporins, monobactams and penicillins were reported [4]. Thus, carbapenems, one of the 'last resort antibiotics' were often used to treat serious infections caused by ESBL carrying pathogens.

*K. pneumoniae* was first reported to harbor *Klebsiella pneumoniae* carbapenemase (KPC), one of the epidemiologically important carbapenemases first detected in North Carolina, USA in 1996 [5]. It was later identified in outbreaks in USA and was soon detected in many European countries and South America [6]. Initially, it was thought that carbapenemases were attributed to chromosomally encoded beta-lactamases and can only be transferred through clonal spread. However, plasmid encoded *bla*_IMP-1_ and *bla*_KPC-1_ reported in the 1990s [7] confirmed the presence of these resistance genes in mobile genetic elements which implicates potential for resistance transmission through horizontal gene transfer.

The first report of carbapenem resistant *K. pneumoniae* in Malaysia was an imipenem resistant strain isolated from blood culture of a 42-year old woman in 2004 [8]. To date, carbapenemases harbored by *K. pneumoniae* isolated in Malaysia identified using Pubmed search were NDM-1 [9, 10], OXA-232 [9] and IMP-4 [11].

In this study, we investigated the genotypic characteristics of carbapenem resistant *K. pneumoniae* isolated from patients in a tertiary teaching hospital in Malaysia. Their association with Tn*4401* and the loss of porin as well as their subtypes were identified. Further risk factors associated with in-hospital mortality rate were also evaluated.

## Methods

### Bacterial isolates

A total of seventeen *Klebsiella pneumoniae* strains isolated within an eight-month period in 2013 since the first report of carbapenem resistant Enterobacteriaceae in this tertiary teaching hospital were revived from stock cultures. Antimicrobial susceptibility testing (AST) was conducted and carbapenemase production of the strains was detected by the Modified Hodge Test (MHT) (*K. pneumoniae* ATCC® BAA-1705 and *K. pneumoniae* ATCC® BAA-1706 as MHT positive and negative reference strain respectively). Clinical records of the patients from whom strains were isolated were retrieved from the hospital database and ethics approval (MEC:1059.15) was obtained from University of Malaya Medical Centre (UMMC) Ethics Committee prior to the start of this study.

### Determination of minimal inhibitory concentration (MIC)

These seventeen strains which had been previously detected as resistant to imipenem and/or meropenem based on Clinical and Laboratory Standards Institute (CLSI) guidelines [12] by the hospital's diagnostic microbiology lab were subjected to E-test (bioMérieux, USA) using *E. coli* ATCC® 25922 as the quality control strain for susceptibility testing. The MIC values (μg/ml) for tigecycline, tetracycline, cefoxitin, cefotaxime, ceftazidime, ceftriaxone, cefepime, imipenem, meropenem, ertapenem, gentamicin, tobramycin, amikacin, aztreonam, ciprofloxacin, levofloxacin and colistin were determined. The results were interpreted using CLSI guidelines [12] while susceptibility to colistin and tigecycline were interpreted according to European Committee on Antimicrobial Susceptibility Testing (EUCAST) breakpoints [13]. In brief, imipenem and meropenem resistance were defined as MIC ≥4 μg/ml while ertapenem resistance was defined as MIC ≥2 μg/ml.

### Pulsed-field gel electrophoresis (PFGE)

The clonal relatedness of these seventeen strains was determined using PFGE. Briefly, plugs containing whole genomic DNA of *K. pneumoniae* strains were digested with *Xba*I. The DNA fragments were separated in a PFGE CHEF-DR III system (Bio-Rad, UK). PFGE conditions of *Xba*I macrorestriction analysis were 6Vcm^-1^ for 20 hours, with pulse times ranging from 5 s to 30s at a temperature of 14 °C and at an angle of 120°. The banding patterns were analyzed using BioNumerics software and similarity >85% upon dendrogram analysis was considered to represent the same PFGE pattern groups.

### Multilocus sequence typing (MLST)

Seven housekeeping genes of *K. pneumoniae* namely, *gapA, infB, mdh, pgi, phoE, rpoB* and *tonB* were sequenced and analyzed using Institut Pasteur MLST and whole genome MLST database for *K. pneumoniae*, http://bigsdb.web.pasteur.fr/klebsiella/klebsiella.html. Each locus was assigned an allele number and the sequence type (ST) of each strain was determined based on the allelic profile generated using the allele numbers of the seven loci.

### Detection of carbapenemases, AmpC beta-lactamases, extended-spectrum beta-lactamases (ESBL) and outer membrane porin genes

Carbapenemase genes such as KPC [14], OXA-48 [15], IMP, VIM [16], NDM [6], NMC, IMI, SIM, SPM [7], GIM [17], AmpC beta-lactamases (CMY, DHA, ACC, FOX) [18] as well as other ESBL associated genes such as TEM, SHV [18], OXA-1 [19], OXA-9 [15], CTXM-1, CTXM-2 [20] were amplified via PCR. The amplified products were sequenced and gene types were compared to the nearest gene homology using a BLAST search. The presence of porin associated genes namely ompK35, ompK36 and ompK37 [21] were also determined by PCR.

### Determination of genetic elements of Tn*4401*

Genetic elements of Tn*4401*, a 10 kb Tn*3* based transposon commonly found to harbor *bla*_KPC_ was determined as previously described [14, 22]. The unconserved region located between IS*Kpn*7 and *bla*_KPC_ gene was also amplified to determine the isoforms of Tn*4401*.

### Statistical analyses

Risk factors associated with in-hospital mortality were compared statistically depending on the level of measurement. Specifically nominal parameters (eg. mechanical ventilation, resistance to antibiotics, etc) were compared using chi-square or Fishers' exact test as appropriate while continuous variables (age and length of hospitalization) were analyzed using either student *t* test or Mann Whitney U test depending on data normality. *P* values less than 0.05 were deemed as statistically significant. Multiple logistic regression was not included in this study due to the small sample size. Continuous variables were summarized as mean ± standard deviation or median (inter quartile range) for both normal and non-normal distribution respectively.

## Results

### Overview of carbapenem resistant *Klebsiella pneumoniae* cases

**Fig. 1:**
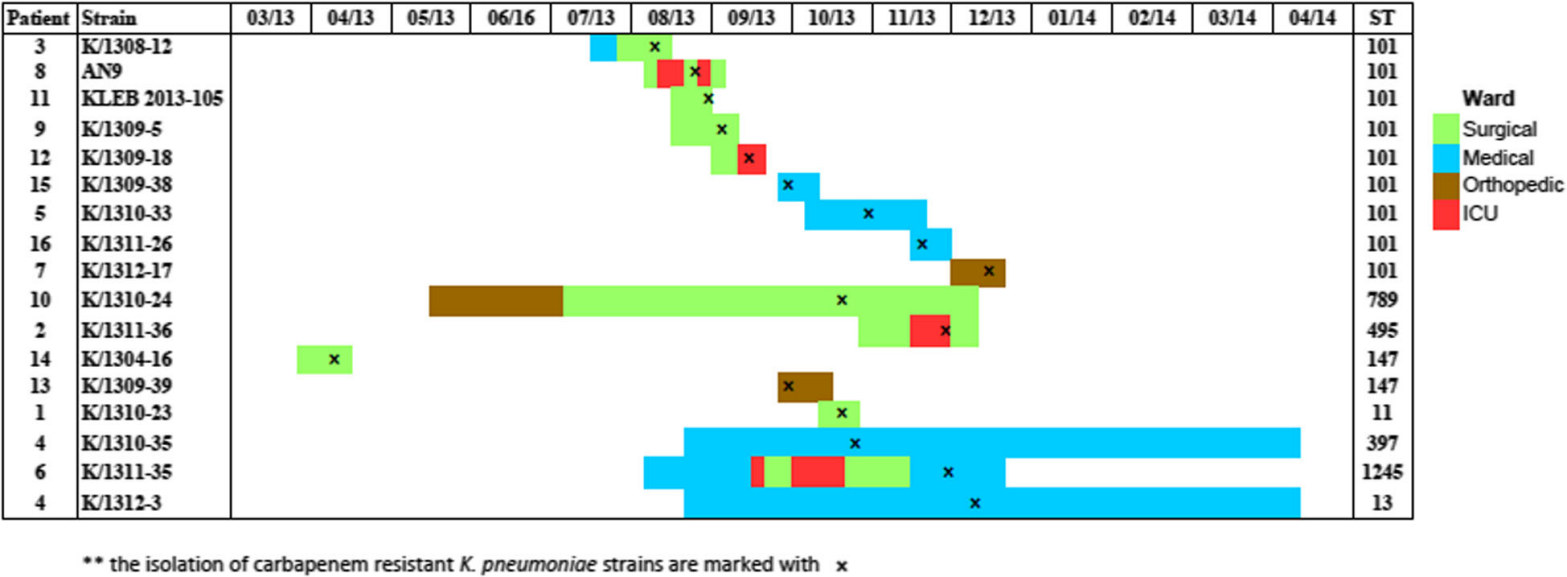
Time-line of events during patient admission period.

The first case of carbapenem resistant *K. pneumoniae* in this study was detected from blood culture of a 57-year-old male patient in April 2013 in this 1,000 bed tertiary teaching hospital in Kuala Lumpur, Malaysia. Since then, no new cases were reported until another carbapenem resistant *K. pneumoniae* isolation from peritoneal drainage of a 67-year-old male patient in August 2013. In the following weeks, an average of one new carbapenem resistant case was detected every 2 weeks and as of 19 December 2013, a total of seventeen strains which were resistant to imipenem and/or meropenem were identified from blood (n = 4), urine (n = 4), swabs (stoma, perirectal, foot) (n = 3), drainage fluids (percutaneous transphetic biliary drainage (PTBD), peritoneal) (n = 2), tracheal secretion (n = 1), tracheal aspirate (n = 1), sputum (n = 1) and tissue (n = 1)). The time-line of events during in-patient admission period including the wards stayed and carbapenem resistant *K. pneumoniae* strains isolation from the sixteen patients from March 2013 to April 2014 was illustrated in Fig. 1. Four of the patients stayed in surgical wards, four in medical wards, two in orthopedic wards and six others moved from different wards. A total of four patients stayed in ICU during their admission period.

These strains were the first carbapenem resistant *K. pneumoniae* isolate from each patient with the exception of two strains which were isolated from a 60-year-old male patient. The first strain isolated from this patient was in October 2013 from tracheal aspirate. Subsequently, we did not identify carbapenem resistant *K. pneumoniae* from respiratory samples with the Vitek2 automated susceptibility testing system in 2013 while urine samples had no growth from October until November 2013. However, carbapenem resistant *K. pneumoniae* was detected in this patient's urine samples in December 2013.

This study was conducted in a multiracial community in Malaysia which comprises of three major races namely Malays, Chinese and Indians. There were nine male patients and seven female patients. The mean age of the patients was 66.13 years where the youngest patient was 48-years-old while the eldest was 85-years-old (Table 1). The average length of hospitalization prior to strain isolation was 65.18 days. Two carbapenem resistant *K. pneumoniae* strains (K/1309-38 and K/1311-26) were isolated from urine and blood respectively from two different patients within 48 hours of admission and one of the patients (K/1309-38) had came into contact with healthcare facilities in the past 30 days for endoscopic procedures. All patients in this study had co-morbidities such as diabetes mellitus, hypertension, chronic kidney disease and solid organ tumors. Diabetes mellitus and hypertension were the predominant non-communicable diseases identified in these patients.

**Table 1:** Clinical cases of patients from whom carbapenem resistant *Klebsiella pneumoniae* was isolated

Isolate	Gender/ Age (years)	Underlying diseases	Isolation site	Length of hospitalization prior to strain isolation (days)	Stay in ICU (days)	Undergone surgical procedure	Use of medical devices^a^	Prior antibiotic exposure (90 days prior to strain isolation)	Empiric antibiotic	Targeted antibiotic	Colonization / Infection	In-hospital death
K/1310-23	M / 85	SOT	Blood	5	0	No	MV, Cath	XM, CI	TZP	CT	Infection	Yes
K/1311-36	M / 72	HPT	Drainage fluid	37	17	Yes	MV, Cath	CP, IP^b^, PM, CLX, TX, CI, TZP, VA, MP^b^	VA, MP	MP, CT	Infection	Yes
K/1308-12	M / 67	DM, HPT	Drainage fluid	21	0	Yes	MV, Cath	CP, MTZ	CI	IP, CT	Infection	No
K/1312-3	M / 60	DM, HPT	Urine	110	0	Yes	MV, Cath	P, TZP, IP^b^	XM, IP	IP^d^	Colonization	Yes
K/1310-33	F / 63	DM, HPT, CKD	Blood	24	0	Unknown	Cath	CFZ, TZ, VA, IP^b^	CT	CT	Infection	No
K/1311-35	M / 71	DM, HPT	Sputum	108	5	Yes	Unknown	VA, CI, MP^b^	TZ	Nil	Colonization	No
K/1312-17	F / 77	DM	Urine	16	0	Yes	Cath	CLX, AMS	CI	Nil	Colonization	No
AN9	F / 62	HPT	Urine	16	15	No	MV, Cath	CP, MTZ, AMS	TZP, IP^b^	IP^d^	Colonization	No
K/1309-5	F / 48	DM, HPT	Tissue	21	0	Yes	MV, Cath	AMS, AMC, TZP, IP^b^	IP^b^	Nil	Colonization	No
K/1310-24	F / 69	DM, HPT	Swab	155	0	Yes	MV, Cath	VA, MTZ, CT, IP^c^, CI, XM, TZP	VA, TZP	VA, TZP	Colonization	No
KLEB-2013-105	F / 74	SOT	Swab	10	0	Yes	MV, Cath	OTC	AMS	Nil	Colonization	No
K/1309-18	F / 74	HPT	Tracheal secretion	10	11	Yes	MV, Cath	CP, MTZ	TZP	Nil^e^	Infection	Yes
K/1309-39	M / 60	DM, HPT, CKD	Swab	3	0	Yes	Cath	AMS	AMS	IP, CT	Infection	No
K/1304-16	M / 57	DM, HPT	Blood	12	0	Yes	Cath	CI, TX	TZP	IP, CT	Infection	No
K/1309-38	M / 62	DM, SOT	Urine	0	0	Yes	MV, Cath	CI, CP, MTZ	TX	IP, CT	Infection	Yes
K/1311-26	F / 57	DM, HPT, SOT	Blood	0	0	Unknown	Unknown	AMS, CI	TX	Nil	Colonization	No
K/1310-35	M / 60	DM, HPT	Tracheal aspirate	67	0	Yes	MV, Cath	P, TZP	Nil	Nil	Colonization	Yes

*M* Male, *F* Female, *SOT* Solid organ tumor, *DM* Diabetes mellitus, *HPT* Hypertension, *CKD* Chronic kidney disease, *ICU* Intensive care unit, *MV* Mechanical ventilation, *Cath* catheter, *Unknown* patient's medical files could not be retrieved, *XM* Cefuroxime, *CI* Ciprofloxacin, *TZP* Piperacillin-tazobactam, *CT* Colistin, *CP* Cefoperazone, *IP* Imipenem, *PM* Cefepime, *CLX* Cloxacillin, *TX* Ceftriaxone, *VA* Vancomycin, *MP* Meropenem, *MTZ* Metronidazole, *P* Penicillin, *CFZ* Cefazolin, *TZ* Ceftazidime, *AMS* Ampicillin-sulbactam, *AMC* Amoxicillin-clavulanate, *OTC* Oxytetracycline

^a^mechanical ventilation and catheter; either urinary catheter, central venous catheter or drainage catheter, ^b^carbapenem exposure for more than 7 days, ^c^carbapenem exposure for 3 -7 days, ^d^carbapenem administered due to other infections, ^e^patient passed away before targeted therapy was initiated

All 16 patients had been administered antibiotics over a period of 90 days prior to carbapenem resistant *K. pneumoniae* strain isolation. Cephalosporins and beta-lactam/beta-lactamase inhibitor combinations were the two most common classes of antibiotics being administered to them. These patients had been treated empirically with antibiotics such as beta-lactam/beta-lactamase inhibitors, carbapenems, cephalosporins, ciprofloxacin, vancomycin and colistin prior to carbapenem resistant *K. pneumoniae* identification (Table 1).

Eight patients (47.06%) in this study were infected with carbapenem resistant *K. pneumoniae* while others were considered to be colonized. Of the eight infected patients, one of them had changed their antimicrobial regimen to colistin while five others were given carbapenem and colistin combination. One patient (12.5%) died before changing therapy and another continued with colistin therapy.

Risk factors such as surgical procedures undergone, usage of mechanical ventilation and the usage of catheter were also taken into consideration. However, based on univariate analyses, only the usage of mechanical ventilation (*P* = 0.043) shows significant effect to in-hospital mortality rate (Table 2).

**Table 2:** Characteristics of carbapenem resistant *Klebsiella pneumoniae* associated with in-hospital mortality.

Characteristics	Survivors (*n* = 11)	Non-survivors (*n* = 6)	*P*
Age (years)	64.09 ± 2.57	68.83 ± 4.09	0.351^b^
Ethnicity			
Malay	2	2	0.317^c^
Chinese	6	3	0.317^c^
Indian	3	1	1.000^c^
Gender			
Male	4	5	0.111^c^
Female	7	1	0.034^c^
Underlying diseases			
Hypertension	9 (82%)	4 (67%)	0.584
Diabetes mellitus	9 (82%)	3 (50%)	0.280
Solid organ tumor	2 (18%)	2 (33%)	0.584
Chronic kidney disease	2 (18%)	0 (0%)	0.515
Site of isolation			
Blood	3 (27.3%)	1 (16.7%)	0.515
Swabs	3 (27.3%)	0 (0%)	0.515
Urine	2 (18.2%)	2 (33.3%)	0.58
Drainage fluids	1 (9.1%)	1 (16.7%)	1.000
Sputum	1 (9.1%)	0 (0%)	1.000
Tissue	1 (9.1%)	0 (0%)	1.000
Tracheal aspirate	0 (0%)	1 (16.7%)	0.353
Tracheal secretion	0 (0%)	1 (16.7%)	0.353
Length of hospitalization (days)	35.09 ± 14.88	38.17 ± 17.65	1.000^d^
Stay in ICU	2 (18%)	2 (33%)	0.584
Undergone surgical procedure	8 (73%)	5 (83%)	1.000
Used ventilation devices	5 (45%)	6 (100%)	**0.043**
Used catheter	9 (82%)	6 (100%)	0.515
Antibiotic prescribed 90 days prior to strain isolation			
Cephalosporins	6 (55%)	4 (67%)	1.000
Penicillins & beta-lactam/beta-lactamase inhibitors	6 (55%)	3 (50%)	1.000
Carbapenems	4 (36%)	2 (33%)	1.000
Colistin	1 (9%)	0	1.000
Others (ciprofloxacin, vancomycin, metronidazole & oxytetracycline)	8 (73%)	4 (67%)	1.000
Empiric treatment			
Beta-lactam/beta-lactamase inhibitors	5 (46%)	2 (33%)	1.000
Cephalosporins	2 (18%)	2 (33%)	0.584
Carbapenems	2 (18%)	2 (33%)	0.584
Ciprofloxacin	2 (18%)	0	0.515
Vancomycin	1 (9%)	1 (17%)	1.000
Colistin	1 (9%)	0	1.000
Targeted therapy			
Carbapenem + Colistin	3 (27%)	2 (33%)	1.000
Colistin	1 (9%)	1 (17%)	1.000
Carbapenem (Meropenem / Imipenem)	1 (9%)	1 (17%)	1.000
Vancomycin & Piperacillin-tazobactam	1 (9%)	0	1.000
Nil^a^	5 (46%)	2 (33%)	1.000
Colonization / Infection			
Colonization	7 (63%)	2 (33%)	0.162
Infection	4 (36%)	4 (67%)	0.162
Resistance to antibiotics (MIC)			
Imipenem	10 (91%)	2 (33%)	**0.04** ^**c**^
Meropenem	9 (82%)	6 (100%)	0.515
Ertapenem	9 (82%)	5 (83%)	0.232^c^
Carbapenemases			
OXA-48	8 (73%)	4 (67%)	1.000
KPC-2	5 (45%)	3 (50%)	1.000
IMP-8	2 (18%)	0 (0%)	0.515
NMC-A	0 (0%)	2 (33%)	0.110
NDM-1	1 (9%)	0 (0%)	1.000
AmpC beta-lactamases			
FOX-7	11 (100%)	6 (100%)	NA
ESBL			
CTXM-2	11 (100%)	6 (100%)	NA
SHV	11 (100%)	6 (100%)	NA
OXA-1	7 (64%)	2 (33%)	0.335
CTXM-15	6 (55%)	4 (67%)	1.000
TEM	6 (55%)	3 (50%)	1.000
OXA-9	6 (55%)	1 (17%)	0.304^c^

Values are expressed as *n* (%) except where otherwise noted.

^a^targeted therapy was not administered as strain was colonizer or patient passed away before targeted therapy was initiated

*P-*values were obtained using Fishers' exact test unless noted otherwise. ^b^ *P-*values obtained using student *t* test, ^c^ *P-*values obtained using chi-square, ^d^ *P-*values obtained using Mann Whitney U test. The significant *P* values were highlighted in bold.

### Determination of minimal inhibitory concentration (MIC)

The antibiotic profiles were tabulated in Table 3. Briefly, all strains were resistant to tetracycline (two strains were intermediate) but only one strain was resistant to tigecycline. Meanwhile, all strains were sensitive to colistin. Eight strains (47.06%) were resistant to all cephalosporins and all strains showed resistance to at least one carbapenem (MIC = 4 - >32 μg/ml) in this study. Resistance to imipenem was significantly associated with in-hospital mortality (*P* = 0.043) (Table 2). It was also noted that two strains (K/1311-35 and K/1304-16) were resistant to imipenem (MIC = 4 - 6 μg/ml) but were sensitive to meropenem and ertapenem while one strain (K/1312-3) was sensitive to imipenem but resistant to meropenem and ertapenem (MIC = 6 - 16 μg/ml). Furthermore, three strains (17.65%) were resistant to all aminoglycosides (MIC = 24 - >256 μg/ml for gentamicin and tobramycin; MIC = 64 - >256 μg/ml for amikacin) while thirteen strains (76.47%) were resistant to all fluoroquinolones (MIC = 8 - >32 μg/ml).

**Table 3:** MIC for the seventeen carbapenem resistant *Klebsiella pneumoniae* strains

Strains	Antibiotics (MIC range in μg/ml)																
	^a^TGC (≤1: S, >2: R)	TC (≤4: S, 8 = I, ≥16: R)	FX (≤8: S, 16 = I, ≥32: R)	CT (≤1: S, 2 = I, ≥4: R)	TZ (≤4: S, 8 = I, ≥16: R)	TX (≤1: S, 2 = I, ≥4: R)	PM (≤2: S, 4-8 = SDD, ≥16: R)	IP (≤1: S, 2 = I, ≥4: R)	MP (≤1: S, 2 = I, ≥4: R)	ETP (≤0.5: S, 1 = I, ≥2: R)	GM (≤4: S, 8 = I, ≥16: R)	TM (≤4: S, 8 = I, ≥16: R)	AK (≤16: S, 32 = I, ≥64: R)	AT (≤4: S, 8 = I, ≥16: R)	CI (≤1: S, 2 = I, ≥4: R)	LE (≤2: S, 4 = I, ≥8: R)	^a^CT (≤2: S, >2: R)
K/1310-23	2	>256	>256	>256	>256	>256	>256	1.5	6	8	>256	>256	32	>256	>32	>32	1.5
K/1311-36	4	256	>256	256	>256	>256	>256	1.5	>32	6	0.094	0.19	0.5	>256	0.047	0.25	0.19
K/1308-12	1	>256	>256	>256	>256	>256	>256	>32	>32	>32	>256	48	24	>256	>32	>32	0.38
K/1312-3	2	>256	2	>256	>256	>256	24	1	6	16	0.38	1.5	256	48	>32	8	0.125
K/1310-33	0.75	>256	128	>256	48	>256	>256	>32	>32	>32	>256	>256	64	>256	>32	>32	0.06
K/1311-35	0.38	6	1.5	4	32	8	0.38	6	0.64	0.094	0.125	0.125	3	24	0.125	0.5	0.19
K/1312-17	0.75	>256	128	6	1	4	16	>32	>32	>32	>256	128	3	0.125	>32	>32	0.125
AN9	1.5	>256	48	4	0.38	2	1	>32	>32	>32	>256	8	3	0.19	>32	>32	0.125
K/1309-5	0.38	>256	>256	>256	128	>256	>256	2	>32	>32	>256	>256	24	>256	>32	>32	0.19
K/1310-24	0.5	>256	2	0.75	0.19	1.5	1.5	6	>32	6	0.19	0.19	1	0.047	0.5	0.5	0.125
KLEB 2013-105	0.19	>256	64	>256	128	>256	>256	>32	>32	>32	96	>256	24	>256	>32	>32	0.25
K/1309-18	0.75	>256	>256	6	0.5	3	2	>32	>32	>32	>256	6	4	0.25	>32	>32	0.25
K/1309-39	0.25	>256	>256	>256	>256	>256	64	12	>32	16	>256	>256	>256	>256	>32	>32	0.125
K/1304-16	1	>256	3	64	192	>256	24	4	0.064	0.125	>256	>256	>256	192	>32	>32	0.125
K/1309-38	0.19	>256	>256	>256	128	>256	>256	4	>32	>32	>256	24	12	>256	>32	>32	0.38
K/1311-26	1	>256	6	1	0.75	1	3	4	>32	24	>256	64	24	0.125	>32	>32	0.38
K/1310-35	0.25	6	3	0.75	0.094	1	0.125	3	>32	0.75	0.125	0.25	2	0.047	0.023	0.125	0.19

*TGC* Tigecycline, *TC* Tetracycline, *FX* Cefoxitin, *CT* Cefotaxime, *TZ* Ceftazidime, *TX* Ceftriaxone, *PM* Cefepime, *IP* Imipenem, *MP* Meropenem, *ETP* Ertapenem, *GM* Gentamicin, *TM* Tobramycin, *AK* Amikacin, *AT* Aztreonam, *CI* Ciprofloxacin, *LE* Levofloxacin, *CT* Colistin, *S* Sensitive, *I* Intermediate, *R* Resistant, *SDD* Susceptible-dose dependent

All MIC values were interpreted using CLSI, 2015 guidelines unless noted otherwise. ^a^MIC values were interpreted based on EUCAST breakpoints, 2016

### Molecular typing via PFGE & MLST

**Fig. 2:**
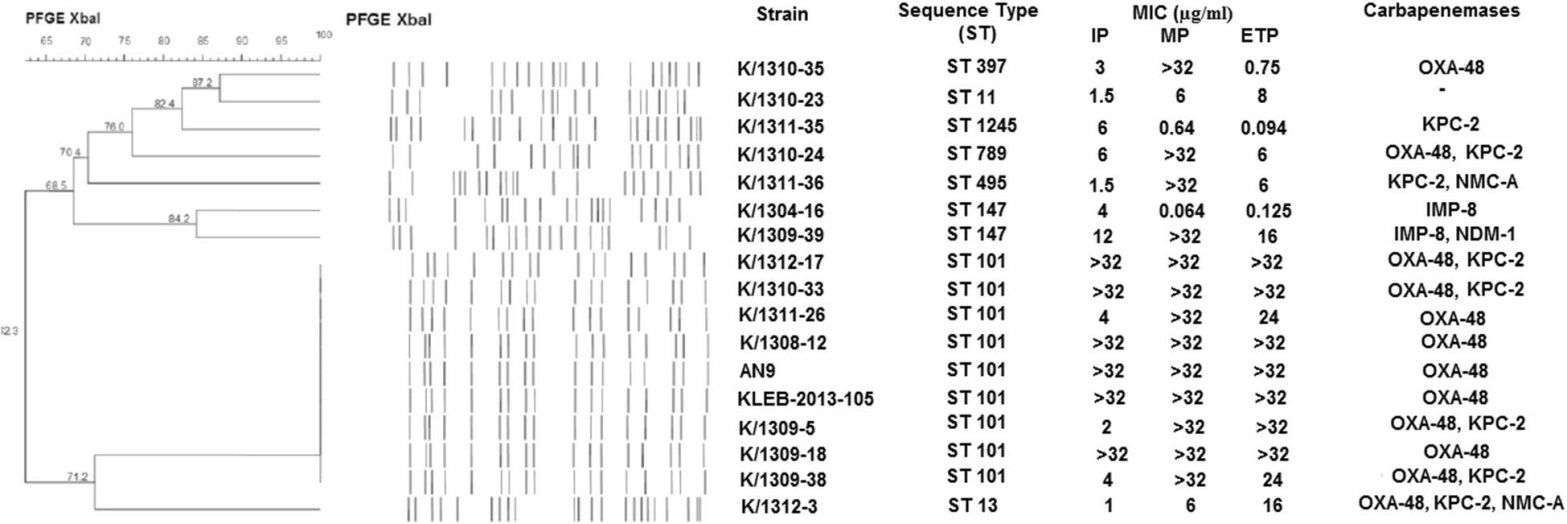
Dendogram of PFGE banding patterns of carbapenem resistant *Klebsiella pneumoniae* and their sequence type (ST) determined via MLST together with their antibiotic and genotypic profile. IP: imipenem, MP: meropenem, ETP:ertapenem

Based on the PFGE patterns obtained, the seventeen strains formed nine different pulsotypes while MLST analysis showed eight STs (Fig. 2). Nine strains affiliated with the predominant sequence type, ST101 demonstrated similar PFGE patterns (Dice index >85% homology) suggesting close intra ST-type genomic similarity. Five of the ST101 strains were isolated within a week apart from one another in August until September 2013 from patients who had stayed in surgical wards. Two weeks later, this sequence type was isolated from one patient in the medical ward and in the following months from two patients in medical wards and one patient from the orthopedic ward. It was also noted that three other sequence types, ST789, ST11 and ST495 were isolated from patients who had stayed in surgical wards two months after the first ST101 isolation and one of the patients had stayed in the surgical ward in August 2013. All ST101 strains in this study were highly resistant to meropenem (MIC >32 μg/ml) and ertapenem (MIC = 24 - >32 μg/ml). High MIC was also observed for imipenem (MIC >32 μg/ml) with the exception of three strains which had lower MICs ranging from 2 μg/ml (intermediate) to 4 μg/ml (resistant).

Similarly, two strains (K/1304-16 and K/1309-39) (Dice index = 84.2% homology) from the same sub-cluster were categorized into the same sequence type, ST147. However, two other strains, K/1310-35 and K/1312-3 which were isolated two months apart from a 60-year-old male patient exhibited distinct PFGE pattern and sequence type (ST397 and ST13 respectively); indicating low relatedness.

### Detection of carbapenemases, AmpC beta lactamases, extended spectrum beta lactamases (ESBL) and porin protein genes

AmpC (*bla*_FOX-7_) and ESBLs (*bla*_SHV_, *bla*_TEM,_ *bla*_CTXM-2_ *bla*_CTXM-15_, *bla*_OXA-1_, *bla*_OXA-9_) were present in all 17 strains while carbapenemases (*bla*_KPC-2_, *bla*_OXA-48_, *bla*_IMP-8_, *bla*_NDM-1,_ *bla*_NMC-A,_) were detected in only 16 strains where *bla*_OXA-48_ was the dominant carbapenemase (Table 4). All strains also exhibited the presence of three outer membrane porins (ompK35, ompK36, ompK37) with the exception of K/1310-24 where ompK36 was absent. This strain, K/1310-24 also showed the highest resistance towards imipenem (MIC = 6 μg/ml), meropenem (MIC = >32 μg/ml) and ertapenem (MIC = 6 μg/ml) among the group of six strains made up of sequence type other than ST101 and ST147.

**Table 4:** PCR results of gene specific primers depicting Carbapenemase, AmpC, ESBL, porin protein and Tn*4401*-like elements in carbapenem resistant *Klebsiella pneumoniae* strains

Strain	Sequence Type (ST)	Carbapenemase	AmpC	ESBL	Porin protein	Genetic elements surrounding Tn*4401*					
						IRL	*tnpA*	IS*Kpn*7	IS*Kpn*6	Variable region	IRR
K/1310-23	11	-	FOX-7	SHV-11, CTXM-15, CTXM-2	OmpK35, OmpK36, OmpK37	-	-	-	-	-	-
K/1311-36	495	KPC-2, NMC-A	FOX-7	TEM-135, SHV-12, CTXM-15, CTXM-2	OmpK35, OmpK36, OmpK37	+	-	+	-	+	+
K/1308-12	101	OXA-48	FOX-7	TEM-1, SHV-28, OXA-1, OXA-9, CTXM-15, CTXM-2	OmpK35, OmpK36, OmpK37	-	-	-	-	-	-
K/1312-3	13	OXA-48, KPC-2, NMC-A	FOX-7	TEM-135, SHV-148, CTXM-15, CTXM-2	OmpK35, OmpK36, OmpK37	-	-	+	-	+	+
K/1310-33	101	OXA-48, KPC-2	FOX-7	TEM-1, SHV-28, OXA-1, OXA-9, CTXM-15, CTXM-2	OmpK35, OmpK36, OmpK37	-	-	+	+	+	-
K/1311-35	1245	KPC-2	FOX-7	SHV-12, CTXM-2	OmpK35, OmpK36, OmpK37	-	+	-	-	-	+
K/1312-17	101	OXA-48, KPC-2	FOX-7	SHV-28, OXA-1, CTXM-2	OmpK35, OmpK36, OmpK37	-	-	-	+	-	-
AN9	101	OXA-48	FOX-7	SHV-28, OXA-1, CTXM-2	OmpK35, OmpK36, OmpK37	-	-	-	-	-	-
K/1309-5	101	OXA-48, KPC-2	FOX-7	TEM-1, SHV-28, OXA-1, OXA-9, CTXM-15, CTXM-2	OmpK35, OmpK36, OmpK37	-	-	+	+	+	-
K/1310-24	789	OXA-48, KPC-2	FOX-7	SHV-121, CTXM-2	OmpK35, OmpK37	-	-	+	-	+	+
KLEB 2013-105	101	OXA-48	FOX-7	TEM-1, SHV-28, OXA-1, OXA-9, CTXM-15, CTXM-2	OmpK35, OmpK36, OmpK37	-	-	-	-	-	-
K/1309-18	101	OXA-48	FOX-7	SHV-28, OXA-1, CTXM-2	OmpK35, OmpK36, OmpK37	-	-	-	-	-	-
K/1309-39	147	IMP-8, NDM-1	FOX-7	TEM-1, SHV-11, OXA-9, CTXM-15, CTXM-2	OmpK35, OmpK36, OmpK37	-	-	-	-	-	-
K/1309-38	101	OXA-48, KPC-2	FOX-7	TEM-1, SHV-28, OXA-1, OXA-9, CTXM-15, CTXM-2	OmpK35, OmpK36, OmpK37	-	-	+	+	+	-
K/1311-26	101	OXA-48	FOX-7	SHV-28, OXA-1, CTXM-2	OmpK35, OmpK36, OmpK37	-	-	-	-	-	-
K/1310-35	397	OXA-48	FOX-7	SHV-1, CTXM-2	OmpK35, OmpK36, OmpK37	+	-	-	-	-	+

*IRL* inverted repeat left, *tnpA* transposase, IS*Kpn*7 insertion sequence 7, IS*Kpn*6 insertion sequence 6, *IRR* inverted repeat right

### Characterization results for Tn*4401*

**Fig. 3:**
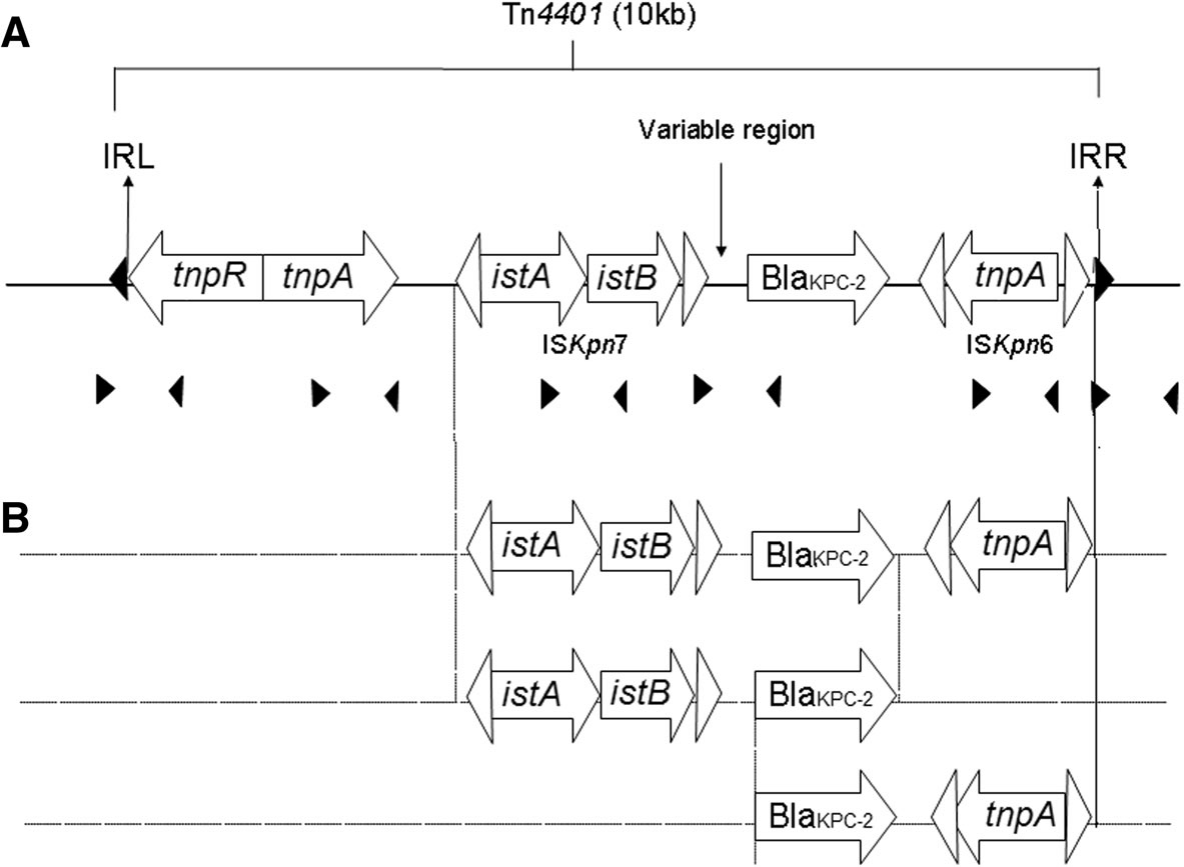
Shaded triangles represent inverted repeats (IR) sequences. Empty triangles represent IRs of insertion sequences, IS*Kpn*6 and IS*Kpn*7. The location of the variable region is as labeled. (Adapted from [14] and [22]). **a**: Schematic representation of intact classical Tn*4401*. **b**: Examples of truncated Tn*4401* detected in this study. IS*Kpn*7, encodes two consecutive open reading frames (ORF); *istA* which encodes a 341-amino-acid putative transposase while *istB* encodes a 259-amino-acid transposition helper protein. *TnpA* encodes a transposase of 1,009 amino acids while *tnpR* is a 1,713 bp resolvase gene

Primer pairs specific for different genetic elements on Tn*4401* were used to deduce the structure of it. A schematic representation of a typical Tn*4401* structure is shown in Fig. 3a. All strains harboring *bla*_KPC-2_ showed the presence of Tn*4401* elements which indicated that *bla*_KPC-2_ gene was carried within Tn*4401* transposon (Table 4).

Isoforms of Tn*4401* were determined based on the fragment size of primer pair hybridizing IS*Kpn*7 and *bla*_KPC;_ upstream of *bla*_KPC_ [22]. All six strains which showed positive amplification were of the same DNA fragment size (703 bp) and they belonged to isoform b where no deletion in the variable region is observed. Among these six strains, one was sensitive, two were intermediate while three exhibited resistance to imipenem (MIC = 4 - >32 μg/ml) but all strains were resistant to meropenem and ertapenem (MIC = 6 - >32 μg/ml). Both flanking sequences of Tn*4401*, inverted repeat left (IRL) and inverted repeat right (IRR) were present in three out of 17 strains (17.65%) while three others (17.65%) showed only one flanking sequence. Complete Tn*4401* was not detected in this study as some elements were absent in all strains. Examples of truncated Tn*4401* detected in this study were shown in Fig. 3b.

## Discussion

Among the seventeen carbapenem resistant *K. pneumoniae* strains investigated, two of them (K/1310-35 and K/1312-3) showed distinct pulsotypes, sequence types and resistotypes, indicating non-clonal relationship despite being isolated from the same patient. During an outbreak investigation of ESBL producing *K. pnemoniae* in a neonatal intensive care unit in Germany, Haller and co-workers [23] found two isolates from the same patient taken 138 days apart to be affiliated with separate clusters on phylogenetic tree. This could indicate the presence of diverse pathogen populations within individuals which complicates treatment. These two strains isolated in this study were colonizers (strains isolated from tracheal aspirate and urine) and this patient died after 229 days of hospitalization. Dautzenberg and co-workers [24] have reported that patients colonized with carbapenem resistant Enterobacteriaceae have higher mortality rates as compared to non-colonized patients due to long hospital stay.

The administration of multiple antibiotics has been reported as a risk factor for carbapenem resistance acquisition [25] and all sixteen patients in this study were given at least one antibiotic in the past 90 days prior to carbapenem resistant *K. pneumoniae* isolation. Five patients had been administered with carbapenems for more than seven days and another for three days while the ten other patients were exposed to other beta-lactams (penicillins, beta-lactam/beta-lactamase inhibitors and cephalosporins), ciprofloxacin, vancomycin, metronidazole, oxytetracycline and colistin. This is agreeable with findings as shown by Patel and co-workers [26] that carbapenem resistance is not attributed only to previous exposure to carbapenem but also exposure to other antibiotics.

One strain, K/1310-23 harbored only AmpC (*bla*_FOX-7_) and ESBLs (*bla*_SHV-11_, *bla*_CTXM-15_ and *bla*_CTXM-2_) without the presence of carbapenemases or loss of porin but it was resistant to meropenem (MIC = 6 μg/ml) and ertapenem (MIC = 8 μg/ml). This may be due to reduced porin expression [27] in the strain or this strain may have harbored rare carbapenemases or variants that were not tested for in this study. Only strain, K/1310-24 was associated with loss of porin; ompK36 and it also harbored carbapenemases (*bla*_OXA-48_ and *bla*_KPC-2_)._._ This strain also exhibited the highest resistance towards carbapenems in the group of strains other than ST101 and ST147 which supports the findings that loss of porin together with the presence of *bla*_KPC-2_ is associated with elevated carbapenem MIC [28].

It was also observed that three strains exhibited varying susceptibility patterns to carbapenems tested in this study. K/1311-35 and K/1304-16 were resistant to imipenem but sensitive to meropenem and ertapenem while K/1312-3 was sensitive to imipenem but resistant to meropenem and ertapenem. This discrepany in resistance pattern often poses a challenge in carbapenem resistant detection especially if either imipenem or meropenem is used as a representative carbapenem in the screening system [29, 30]. Imipenem resistant, meropenem sensitive *Pseudomonas aeruginosa* and imipenem sensitive, meropenem resistant *K. pneumoniae* strains have been previously reported [30, 31]. This difference in susceptibility patterns can be attributed to the variation in outer membrane porin structure and the efflux pumps expression. The eight amino acid sequence deletion on loop L7 of *OprD*, a carbapenem-specific porin in *P. aeruginosa* sufficiently opens the porin channel, allowing optimal penetration of meropenem and increases its activity without affecting the susceptibility of smaller carbapenem molecules such as imipenem [32]. The overexpression of resistance-nodulation-cell division (RND) efflux pump in *P. aeruginosa* has also been reported to cause varying carbapenem MIC levels [33]. Thus, we postulate that there may be similar porin structure and efflux pump variations in *K. pneumoniae* that contribute to this discrepancy in carbapenem resistance.

Overall, PFGE and MLST produced comparable clustering results. The predominant sequence type, ST101 (n = 9) shared the same pulsotype. This characteristic had been reported by Kitchel and co-workers [34] where *K. pneumoniae* strains with ≥ 80% similarity in PFGE patterns strongly agreed with their MLST results. It was observed that ST101 spread occurred in the surgical ward in August 2013 as five of ST101 strains were isolated from five different patients who had stayed in surgical wards during this one month period. However, ST101 was also isolated from three other patients from medical wards and one patient from orthopedic ward in the following months. We postulate that more complicated transmission routes such as asymptomatic patients who were never detected, via health care personnel or medical devices [35] may have contributed to the spread of ST101 to patients from other wards. ST789, ST11 and ST495 were also isolated from three separate patients in surgical wards in October and November 2013 and one of the patients had came into contact with the ST101 harboring patients as they shared the same ward in August and was colonized. Despite being phylogenetically unrelated to ST101, these three strains harbor similar ESBL and carbapenemase genes indicating horizontal gene transfer may occur since these genes are often found on transposons and plasmids [36, 37].

Our finding of ST101 as a predominant strain is similar to the report of ST101 as a predominant *K. pneumoniae* clone in an acute general hospital in Italy [38]. ST101 was reported to be responsible for *bla*_OXA-48_ outbreaks in Spain and Tunisia [36] and harboring *bla*_KPC-2_ in Italy [38] which was comparable to our results where ST101 isolated were *bla*_OXA-48_ with four co-producing *bla*_KPC-2_.

*Bla*_OXA-48_, which was first reported in Turkey [39] was also known to exhibit susceptibility towards extended-spectrum cephalosporins and carbapenems [40] which complicates the detection via routine clinical microbiology laboratory tests. Similarly, K/1310-35 (sensitive to all cephalosporins), K/1310-24 (intermediate to ceftriaxone while being sensitive to all other cephalosporins) and K/1311-26 (susceptible dose dependent (SDD) to cefepime while being sensitive to all other cephalosporins) harbored *bla*_OXA-48_. Interestingly, these three strains also harbored *bla*_SHV_ and *bla*_CTXM_ which was known to hydrolyze cefotaxime and ceftazidime [41] but all three strains were sensitive to both antibiotics in this study.

*Bla*_KPC_ is endemic in northeastern regions in USA, Greece and Israel but cross regional spreading into United Kingdom, Brazil, Sweden, India and China had been reported recently [42]. This gene is often detected on mobile genetic elements such as plasmids and transposons which facilitates its rapid dissemination worldwide [37]. *Bla*_KPC-2_ is generally associated with Tn*4401* isoforms [43] and in this study, Tn*4401*b (no deletion in the variable region) was associated with six *bla*_KPC-2_ harboring strains. This isoform had been reported to exhibit lower resistance to carbapenems as compared to other isoforms [28]. However, such conclusions cannot be made in this study as only one isoform type was detected in this study. To the best of our knowledge, this report presented the first identification of *K. pneumoniae* harboring *bla*_OXA-48_ and *bla*_KPC-2_ in Malaysia.

Two strains, K/1304-16 and K/1309-39 demonstrated slight differences in PFGE banding patterns (Dice homology = 84.2%) despite having the same sequence type, ST147. PFGE is able to detect chromosomal rearrangements [44] which may contribute to differences in their antibiotic and genotypic profiles. K/1309-39 was resistant to imipenem, meropenem and ertapenem (MIC = 12 - >32 μg/ml) while K/1304-16 demonstrated low resistance to imipenem, (MIC = 4 μg/ml) and was sensitive to both meropenem and ertapenem. Furthermore, *bla*_TEM_ carried by both strains was of different gene types. TEM-1 was present in K/1309-39 while TEM-135 was detected in K/1304-16. These two strains can also be distinguished further based on the presence of *bla*_NDM-1_ in K/1309-39 and the presence of inverted repeats (IR) of Tn*4401* in K/1304-16.

A majority of NDM cases reported worldwide were related to travel or hospitalization in the Indian subcontinent such as India and Pakistan [45] and ST147 has been shown to be associated with *bla*_NDM-1_ in India [46]. However, in our study, the *bla*_NDM-1_ positive patient had no record of prior travel outside Malaysia. The presence of *bla*_NDM_ without any association with international travel has also been reported by Rimrang and co-workers [47] which indicated that the NDM gene was acquired locally.

This study is limited by its small sample size as this study only includes the carbapenem resistant *K. pneumoniae* strains which were isolated during an eight months' period since the first carbapenem resistant *K. pneumoniae* isolation in this hospital. Thus, the findings cannot be generalized to a broader population based on this study alone as it may include potential biases. The significance of mortality rates with imipenem resistance as compared to meropenem and ertapenem resistance cannot be firmly drawn since all the studied strains were imipenem and / or meropenem resistant strains. The increased use of imipenem may have contributed to increased imipenem resistance [48] since five patients were administered with imipenem while only two patients were given meropenem. However, our findings that mechanical ventilation is associated with mortality can be justified as it was also reported in other studies [25, 49].

## Conclusions

The emergence of carbapenem resistant *K pneumoniae* harboring various carbapenemases coupled with the presence of transposon and the loss of porin may contribute to the increasing cases being detected. Thus, microbiological, molecular and clinical data of these strains are important to provide information for a better understanding and to facilitate carbapenem resistance control.

## Abbreviations

AST: Antimicrobial susceptibility testing
CLSI: Clinical and Laborartory Standards Institute
ESBL: Extended spectrum beta-lactamase
EUCAST: European Committee on Antimicrobial Susceptibility Testing
ICU: Intensive care unit
MHT: Modified Hodge test
MIC: Minimal inhibitory concentration
MLST: Multilocus sequence typing
PFGE: Pulsed-field gel electrophoresis
PTBD: Percutaneous transphetic biliary drainage
RND: Resistance-nodulation-cell division
SDD: Susceptible dose dependent
ST: Sequence type
